# Measuring Hidden Support for Physical Intimate Partner Violence: A List Randomization Experiment in South-Central Ethiopia

**DOI:** 10.1177/0886260520914546

**Published:** 2020-05-05

**Authors:** Mhairi A. Gibson, Eshetu Gurmu, Beatriz Cobo, María M. Rueda, Isabel M. Scott

**Affiliations:** 1 University of Bristol, UK; 2 Addis Ababa University, Ethiopia; 3 University of Granada, Spain

**Keywords:** intimate partner violence, domestic violence, wife-beating, unmatched count technique, indirect questioning method: violence against women and girls, reporting biases

## Abstract

Understanding how and why physical intimate partner violence (IPV) persists in high-risk communities has proven difficult. As IPV is both sensitive and illegal, people may be inclined to misreport their views and experiences. By embedding a list randomization experiment (LRE), which increases respondent privacy, in a survey of 809 adult Arsi Oromo men and women in rural south-central Ethiopia, we test the reliability of direct questioning survey methods (e.g., used in the Demographic and Health Surveys) for measuring attitudes that underpin the acceptability of IPV. Participants were randomly assigned versions of the survey in which they were asked either directly or indirectly about the acceptability of wife-beating. By comparing responses across these surveys, we identify the extent to which views are being misreported using direct questioning methods, as well as identifying the “true” predictors of continued support for wife-beating. Indirect questioning reveals that almost one third of the sample believe that wife-beating is acceptable. Adults (particularly men) who are less educated (<3 years schooling) or living in households where women do not participate in economic decision making are among those most likely to identify wife-beating as justifiable (>50% endorsement). These individuals, however, are also more inclined to hide their approval when asked directly by an interviewer. That we find high but underreported support for wife-beating among some members of the community demonstrates a clear need to encourage a more open dialogue, to prevent violence toward women remaining undetected and thus unchanged. This finding also raises questions about the accuracy of traditional direct questioning for capturing information on IPV attitudes and norms. Of relevance to policy, we find that wife-beating is entirely absent only among adults with higher levels of education, living in households where decision making is shared between couples.

## Introduction

Physical intimate partner violence (IPV) by men against women has major implications for women’s physical, reproductive, and psychological health, and their economic welfare and that of the wider community (Ellsberg et al., 2008). Estimates of the number of women who have been assaulted by a male partner range from 71% in south-central Ethiopia dropping to 15% in urban Japan (Garcia-Moreno et al., 2006). A key priority for global campaigns seeking to end physical violence toward women is to gain a better understanding of social attitudes toward IPV and the community norms that foster “a culture of violence toward women”. Acceptance of violence toward intimate partners strongly predicts the incidence of violence (Abramsky et al., 2011; Heise & Kotsadam, 2015) and victims’ responses to the violent act, for example, help-seeking behavior (Goodson & Hayes, 2018).

One major challenge for measuring the social attitudes and beliefs that underpin IPV is the sensitivity of the topic (see review in Yount et al., 2014). People may be reluctant to disclose information concerning IPV, due to its illegality or other community responses, for example, victims may be socially stigmatized or fear retaliation from others (Palermo et al., 2013). In contexts where there is greater acceptability of violence, individuals may overstate their support for IPV. Reporting what is perceived to be socially appropriate and acceptable rather than true beliefs (referred to as social desirability bias) may explain the discrepancies found between intimate partners in self-report surveys (e.g., one in three Tanzanian couples disagree about IPV occurrence; Halim et al., 2018; Yount & Li, 2012). Evidence that individuals may feel under different social pressures to misreport their views on physical IPV is suggested by the gender discrepancy in justification for wife-beating reported in the 2016 Ethiopian Demographic Health Survey (DHS). Here, 63% of women, compared with 28% of men, stated that wife-beating is justifiable (Central Statistical Agency [CSA] [Ethiopia] & ICF International, 2016).

To resolve the problem of misreporting, we used a list randomization experiment (LRE). This is a powerful indirect questioning method used to anonymously obtain responses to “sensitive” questions (Glynn, 2013). List randomization (sometimes referred to as “unmatched count technique”) works by aggregating responses to the sensitive question alongside responses to nonsensitive questions, thereby masking the respondent’s response to the sensitive question (further detail is provided in the “Method” section). LRE has been used extensively by political and economic scientists to explore civic issues, including voting turnout (Holbrook & Krosnick, 2009), socially unacceptable attitudes such as racial prejudice (Aronow et al., 2015), and illegal behaviors from shoplifting (Tsuchiya et al., 2007) to wildlife poaching (Nuno & St. John, 2015). There has been a recent sharp uptake of similar indirect questioning methods to explore sensitive health topics including abortion (Moseson et al., 2017) and sexual behavior (Starosta & Earleywine, 2014). A few studies have used the LRE to record physical harassment and violence toward women, but only in urban and/or educated contexts (e.g., Agüero & Frisancho, 2018; Peterman et al., 2018). LRE remains relatively underused in low-income contexts, for example, rural sub-Saharan Africa (SSA), despite growing recognition that the method may have considerable scope to improve understanding on a wide range of topics related to gender-based violence (e.g., female genital mutilation or cutting [FGMC]; De Cao & Lutz, 2018; Gibson et al., 2018).

Here, we employ an LRE to gain more accurate data on attitudes to wife-beating in an at-risk community in Oromia region, south-central Ethiopia, where there is thought to be high but declining support for physical violence by men against women. Directly reported survey data indicate that the percentage of men justifying wife-beating in Oromia has dropped from 80.9% to 28% in less than 5 years (CSA [Ethiopia] & ICF International, 2012, 2016). We investigate the association between acceptance of physical IPV and five key individual characteristics identified in previous analyses using traditional DHS survey data: age, gender, education level, household wealth, and decision-making norms. The extent to which men and women cooperate in decision making about the use of household economic resources is used as an indicator of underlying gender norms, based on prior research, which suggests that women’s participation in economic decision making reflects the degree of control that women can exercise over their own lives (Semenza et al., 2019; Svec & Andic, 2018).

Analyses of previous directly reported survey data reveal that the odds of justifying physical violence are higher for women than men, with decreasing age, decreasing educational attainment, decreasing wealth, and in households where men alone are responsible for economic decision making (Fulu et al., 2013; Tran et al., 2016; Uthman et al., 2009). However, it is unclear whether these results reflect social desirability and reporting biases. For example, it has been suggested that men may be less inclined to openly endorse violence than women due to social stigma or legal implications (Fulu et al., 2013). Women, conversely, may overstate their acceptance of “wife-beating” in contexts where partner violence is relatively normalized (Halim et al., 2018). Indirect questioning studies have indicated that high socioeconomic status is linked with women underreporting their experience of physical violence in urban Peru (Agüero & Frisancho, 2018) and India (Joseph et al., 2017).

By combining an LRE with traditional self-reported methods we will identify (a) “true” views in support of physical IPV that may otherwise be concealed, (b) the “true” predictors of individual variation in these views, (c) the accuracy of traditional directly reported survey methods by comparing differences between directly reported and indirectly reported responses (Glynn, 2013), and (d) whether participants are inclined to overstate or understate their tolerance of IPV, which may give an indication of how social norms and pressures are operating in the population, and the subgroups within.

## Method

### Data Collection

In 2017, a population-based demographic survey was undertaken with 809 Arsi Oromo adults living in a rural subdistrict of Arsi Zone, Southern Oromia. The Arsi Oromo living in this area are Muslim agropastoralists who subsist primarily through maize and wheat cultivation, and some cattle herding. Agricultural land is limited and there are few jobs outside farming (Gibson & Gurmu, 2011, 2012). This population was selected as existing survey data indicated that there has been a dramatic reduction in support for gender-based violence suggestive of increased reporting biases (Gibson et al., 2018). Furthermore, the demographic and health surveys reveal that the percentage of men justifying wife-beating in Oromia region has dropped from 80.9% to 28% in less than 5 years (CSA [Ethiopia] & ICF International, 2012, 2016).

Community members were informed of the existence and nature of the research project during a weekly community meeting, where they were given the opportunity to discuss their involvement in the study. Informed written consent (or fingerprint consent) was obtained from each individual participant in the study. All households in the community (including those who did not take part in the survey) were given a gift of coffee. Research and ethical approval to undertake this study was granted by the Ethics Committees at the University of Addis Ababa and the University of Bristol.

Prior to the main survey, focus group discussions were undertaken to develop the questionnaire: for instance, choosing the items included in the LRE (further details provided below). The survey was then piloted in a neighboring village, and all interviewers received training in the survey protocols. The survey included direct questioning (DQ) on the acceptability of wife-beating, as well as an “indirect” questioning approach (the LRE).

A random sample of 50% of the households in the community were surveyed; these were alternate households selected from a village plan supplied by the local authorities. Within each household, two surveys were completed by a near equal and randomly selected sample of adult male and female, married and unmarried respondents from a household list, resulting in a total sample of 809 adults. The survey was undertaken in the respondent’s house (or within their compound) by a trained same-gender interviewer fluent in the local language, Oromiffa. No other adult was present. Each survey took less than 30 min, each focus group took less than 1 hr. No participant declined the invitation to take part in the survey.

**Figure 1.**
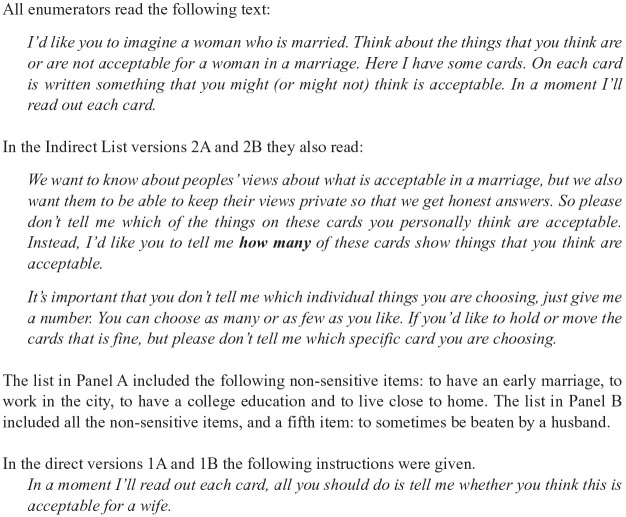
Direct and indirect survey questions.

Respondents were randomly assigned to one of four different versions of the survey. Respondents answered either direct questions (DQ) with or without the sensitive question on wife-beating acceptability (Versions 1B and 1A) or answered an indirect (LRE) list of questions with or without the sensitive question (Versions 2B and 2A). Twenty percent (*n* = 162) answered the direct question, and 80% (*n* = 647) answered the indirect question. This sampling strategy was designed to ensure there were adequate numbers and enough statistical power to perform statistical analyses (*n* = 647), while reducing the relative number of responses to direct questions without the IPV card (four-card control groups), which was included only to test the quality of the indirect (LRE) list. Figure 1 includes a full list of the questions posed in each version of the survey.

For LRE, half of the sample (total *n* = 647) were then randomly assigned to a version of the survey where they were asked to report the number of items on a list of four nonsensitive item cards, which were acceptable for women in marriage (Version 2A; see Figure 1 for details of the questions, and the paragraph below on generating the LRE lists). The other half of the respondents were read the same list of four nonsensitive item cards plus an additional card “to sometimes be beaten by a husband” and asked the same question (Version 2B). An estimate of the proportion of people for whom wife-beating was acceptable was calculated by subtracting the average number reported by the first group of respondents (receiving the four-card list) from the average number reported by the second group (receiving the five-card list). As the respondent understands that the interviewer does not know exactly which card(s) they are choosing, the respondent’s answers to this question are more likely to be undistorted by social desirability bias, and thus be more accurate.

In the direct question (DQ) versions of the survey, half of the sample (total *n* = 162) were randomly assigned to a version with either the four-card list (Version 1A) or the five-card list including the item “to sometimes be beaten by a husband” (Version 1B). In this case, respondents were asked to directly report whether the content of each card read by the interviewer was an acceptable activity or behavior for women in marriage. By comparing these two DQ versions of the survey, it was possible to check for independence of responses, that is, that the sensitive item on wife-beating acceptability did not change people’s tendency to respond “yes” to the other four items on the list. Analyses of the final dataset revealed that this “additional item” test was passed and there was no statistically significant difference in the distribution of responses from direct questions with and without the wife-beating card (Version 1A: *M* = 2.36, *SD* = 0.64; Version 1B: *M* = 2.26, *SD* = 0.60; two-sample Kolmogorov–Smirnov Test: *D* = 0.108, *p* = .736).

By comparing LRE responses (Version 2B) with directly reported (DQ; Version 1B), it was also possible to determine the direction of social influences on reporting (Glynn, 2013), that is, levels and variation in misreporting of views on the acceptability of wife-beating. Past studies have found that the predictors of sensitive items measured with the LRE are different from the predictors of those measured with direct self-reports. For example, in a study of views on female genital cutting (FGC), we found that older individuals report less support for the practice than younger individuals when questioned directly, but the pattern is reversed when questioned indirectly using LRE—indicating that the older age group are more inclined to underreport their continued support for the practice in surveys using traditional self-report questioning methods (Gibson et al., 2018).

### Generating Lists for the LRE

In this study, the list was generated via focus group discussions conducted during a piloting stage. Group members were asked to report popular local views regarding the characteristics of wives, which generated an extended list of potential items, from which four were selected for inclusion in the survey. The final four items were selected to minimize the chance of floor and ceiling effects—that is, of participants selecting either all or none of the items—as this could compromise anonymity by allowing the interviewer to infer that the sensitive trait had/had not been selected. One item was expected to be unpopular (early marriage), one item was expected to be popular (education), and two items were expected to be incompatible (work in the city and live close to home). Expectations regarding the popularity of different items were confirmed in the final data set, with low levels of floor/ceiling effects being observed. Less than 1% of respondents selected all or none of the list items in the list (Gibson et al., 2018).

### Statistical Analyses

Analyses were performed using freely available R software for list randomization analyses (Blair & Imai, 2010). To contrast the proportions between the DQ method and LRE, and for subgroups (in both DQ and LRE methods), we used a contrast of equal proportions (Wolter & Preisendörfer, 2013). We also undertook multivariate analyses using generalized linear regression models developed by Blair and Imai (Blair & Imai, 2010, 2012; Imai, 2011). These multivariate analyses have not been included in this article, as none of the tested models fitted well. This may be due to small sample sizes in some subgroups (see Tables 2 and 3). It also represents a challenge for the methodology; LRE does increase respondent privacy, but it also requires large sample sizes.

## Results

A total of 809 adults were included in the survey and analyses; this included an almost equal number of men and women (403 men and 406 women). To identify whether wife-beating acceptance was associated with individual gender, age group, education level, perceived household wealth group, and household level of female economic empowerment, the sample was divided into subgroups. Two groups were created based on age: 18 to 25 years and 26+ years, and two equally sized groups based on completed years of school (≤3 years and ≥4 years), and two groups based on perceived household wealth score: “high wealth” (scores 1–5) and “low wealth” (scores 6–10). Categories were created to identify gender equity in household decision making: “low gender equity” where men alone made economic decisions and “high gender equity” where economic decisions were made jointly by men and women. Table 1 provides a summary of the characteristics of the sample, as well as a breakdown of estimates of wife-beating acceptance according to question methodology (direct [DQ] vs. indirect [LRE]) and each individual trait.

**Table 1.** A Comparison of DQ and LRE Estimates by Subgroup.

Respondents	*N*	DQ Estimate (*SE*)	LRE Estimate (*SE*)	*p* Value LRE > DQ	*p* Value LRE ≠ DQ
All	809	0.18 (0.005)	0.28 (0.002)	.06	.12
Male	403	0.15 (0.009)	0.32 (0.005)	.06	.12
Female	406	0.20 (0.010)	0.26 (0.004)	.27	.54
Young (18–25 years)	245	0.08 (0.010)	0.26 (0.007)	.06	.12
Older (26+ years)	564	0.22 (0.008)	0.30 (0.003)	.20	.39
Low education (0–3 years)	440	0.16 (0.010)	0.40 (0.004)	.004**	.009**
High education (4+ years)	369	0.19 (0.009)	0.17 (0.005)	.59	.82
Perceived wealth rank					
Higher (score 1–5)	289	0.09 (0.009)	0.35 (0.006)	.007**	.02*
Lower (score 6–10)	520	0.23 (0.009)	0.24 (0.003)	.44	.87
Female economic empowerment					
Male makes all economic decisions	354	0.15 (0.010)	0.45 (0.005)	.002**	.005**
Joint male–female economic decisions	455	0.20 (0.009)	0.17 (0.004)	.60	.80

*Note.* DQ = direct questioning; LRE = list randomization experiment.

* *p* ≤ .05. **p ≤ .01. ***p ≤ .001.

### Direct Versus Indirect (LRE) Questioning Methods

Responses from the LRE indicate that some, but not all people, are privately more supportive of wife-beating than they are prepared to admit openly through DQ methods. When asked directly, only 18% (95% confidence interval [CI] = [9, 26]) of all respondents reported wife-beating as acceptable, whereas the indirect list of responses indicates that “true” support for the behavior is at 28% (95% CI = [17, 40]). However, the differences between contrasts is not statistically significant (contrast LRE ≠ DQ, *p* = .12).

### Individual Characteristics of Respondent

Univariate analyses reveal that men and women report similar levels of acceptance of wife-beating when asked directly, 15% (95% CI = [4, 26]) and 20% (95% CI = [8, 32]), respectively, *p* = .56, or asked indirectly using the LRE list, men: 32% (95% CI = [14, 50]), and women: 26% (95% CI = [13, 39]), *p* = .58. A comparison of LRE and DQ estimates suggests that men but not women conceal their acceptance of wife-beating when questioned directly, 15% rising to 32% among men in response to the list; however, this difference is not statistically significant (contrast LRE ≠ DQ, *p* = .12).

When asked directly, younger individuals (<26 years) report lower endorsement of wife-beating than older (≥26 years), 8% (95% CI = [0, 18]) and 22% (95% CI = [11, 33]), at borderline significance, *p* = .05. LRE estimates, however, reveal no difference in privately held views between older and younger age groups, 26% (95% CI = [6, 46]) and 30% (95% CI = [17, 43]), respectively, *p* = .74. Furthermore, there are no statistically significant differences between direct question (DQ) and list (LRE) results for both age groups, indicating that age does not strongly influence reporting of support for wife-beating.

Education level has no effect on responses to direct questions. Of those respondents with three or less years in school, 16% (95% CI = [4, 27]) endorse wife-beating, compared with 19% (95% CI = [7, 31]) of those with higher education (4+ years; difference: *p* = .70). LRE responses, however, indicate that “true” support for wife-beating is lower among the more educated group (17%, 95% CI = [0, 34]), compared with the less educated group, 40% (95% CI = [26, 54]), difference: *p* = .04. Less educated individuals are more likely to justify wife-beating in response to the LRE list rather than DQ: 16% (95% CI = [4, 27]) expressing direct support for wife-beating, rising to 40% (95% CI = [26, 54]) using the indirect LRE (difference: *p* = .009). For respondents with higher education, the difference between DQ (19%, 95% CI = [7, 31]) and the indirect LRE list (17%, 95% CI = [0, 34]) is not significant (difference: *p* = .82). These results imply that less educated individuals hold views that are more supportive of wife-beating compared with those who are more educated; however, less educated individuals are also more likely to conceal their support when questioned directly about wife-beating.

High or low perceived wealth ranking of the household does not statistically influence estimates of wife-beating acceptance. The wealthier ranked individuals do not differ from the poorer ranked individuals in both DQ, 9% (95% CI = [0, 19]) and 23% (95% CI = [11, 35]), respectively, *p* = .09, and in the list analyses, 35% (95% CI = [17, 54]) and 24% (95% CI = [11, 38]) respectively, *p* = .35. Indirect methods (LRE) reveal that individuals from wealthier households are more likely to endorse wife-beating than revealed through direct (DQ) methods, 35% (95% CI = [17, 54]) and 9% (95% CI = [0, 19]), respectively, *p* = .02. Estimates from individuals from poorer households do not differ between DQ and LRE, 23% (95% CI = [11, 35]) and 24% (95% CI = [11, 38]), respectively, *p* = .87. These results indicate that although perceived household wealth rank does not influence privately held support, people with greater perceived wealth are more inclined to conceal their support for wife-beating when questioned directly.

Finally, level of female economic empowerment within the household is not strongly associated with responses to direct questions on attitudes to wife-beating. When asked directly, 15% (95% CI = [3, 27]) of individuals living in households where men alone make the economic decisions support wife-beating, compared with 20% (95% CI = [8, 31]) of individuals living in households where decision making is shared (difference: *p* = .56). LRE analyses, however, reveal that privately held endorsement of wife-beating is greater in households where men make all the economic decisions compared with those with joint male–female decision making, 45% (95% CI = [27, 63]) and 17% (95% CI = [4, 31]), respectively (difference: *p* = .02). The discrepancy between responses to DQ versus indirect LRE responses, 15% (95% CI = [3, 27]) and 45% (95% CI = [27, 63]) respectively, *p* = .006, for individuals living households where men alone make the economic decisions, suggests that these individuals are more inclined to conceal their support for wife-beating when questioned directly. Within households where economic decisions are made jointly, estimates for DQ and LRE responses (20%, 95% CI = [8, 31], and 17%, 95% CI = [4, 31], respectively) do not differ (*p* = .80).

### Subgroup Analyses of Wife-Beating Norms

Additional subgroup analyses were undertaken to identify subsections of society viewing wife-beating acceptance as normative. We defined “normative” as being where more than 50% of the subgroup shared the view that wife-beating was acceptable. Table 2 includes a breakdown of these analyses, including contrasts between gender and each of the respondent’s individual traits (age group, education level, perceived wealth score, and level of female economic empowerment). Table 3 includes breakdown of subgroup analyses including education level and each of the other individual traits. No other interactions between the individual traits were found to be statistically significant.

**Table 2.** LRE Subgroup Analyses, *n* = 647, Exploring Interactions Between Gender and Each of the Other Traits.

Respondent		*n*	LRE Estimate (*SE*)		*n*	LRE Estimate (*SE*)	*p* Value
Gender	Interaction			Interaction			
Male	<3 years education	119	0.62 (0.01)	4+ years education	202	0.14 (0.01)	.01**
Male	18–25 years	92	0.36 (0.02)	26+ years	229	0.30 (0.01)	.77
Male	High wealth	122	0.31 (0.01)	Low wealth	199	0.32 (0.01)	.95
Male	Joint decisions	173	0.14 (0.01)	Male-only decisions	148	0.55 (0.01)	.03*
Female	<3 years education	234	0.29 (0.01)	4+ years education	92	0.20 (0.01)	.56
Female	18–25 years	100	0.15 (0.01)	26+ years	226	0.31 (0.01)	.24
Female	High wealth	106	0.41 (0.01)	Low wealth	220	0.19 (0.01)	.10
Female	Joint decisions	192	0.21 (0.01)	Male-only decisions	134	0.34 (0.01)	.33

*Note.* LRE = list randomization experiment.

* *p* ≤ .05. **p ≤ .01. ***p ≤ .001.

**Table 3.** LRE Subgroup Analyses, *n* = 647, Exploring Interactions Between Education and Each of the Other Traits.

Respondent		*n*	LRE Estimate (*SE*)		*n*	LRE Estimate (*SE*)	*p* Value
Education	Interaction			Interaction			
<3 years education	Male	119	0.62 (0.01)	Female	234	0.29 (0.01)	.06
<3 years education	18–25 years	43	0.44 (0.05)	26+ years	310	0.39 (0.01)	.81
<3 years education	High wealth	126	0.51 (0.01)	Low wealth	227	0.34 (0.01)	.26
<3 years education	Joint decisions	194	0.39 (0.01)	Male-only decisions	159	0.40 (0.01)	.93
4+ years education	Male	202	0.14 (0.01)	Female	92	0.20 (0.01)	.74
4+ years education	18–25 years	145	0.21 (0.01)	26+ years	145	0.12 (0.01)	.62
4+ years education	High wealth	102	0.18 (0.01)	Low wealth	192	0.15 (0.01)	.90
4+ years education	Joint decisions	171	0.00 (0.01)	Male-only decisions	123	0.50 (0.01)	.003**

*Note.* LRE = list randomization experiment.

* *p* ≤ .05. **p ≤ .01. ***p ≤ .001.

Our analyses reveal that highest levels of support for wife-beating are found among less educated men, where estimated acceptance levels reach 62% (95% CI = [32, 91]), significantly higher than those found among more educated men, 14% (95% CI = [0, 37]; difference: *p* = .01). High level of wife-beating acceptance is also found for men living in households where they alone made all the economic decisions. In this group, estimates reach 55% (95% CI = [27, 83]) and are significantly different from those of men living in joint decision-making households, which are at 14% (95% CI = [0, 37]; difference: *p* = .03). We find the lowest levels of wife-beating acceptance are among the more educated individuals who also live in households where couples share the economic decision making. None of these individuals endorse wife-beating, compared with 50% (95% CI = [20, 78]) of those who are equally well educated, but live in a household where men alone make the economic decisions (difference: *p* = .003).

### Reasons That Wife-Beating Is Justified

**Figure 2.**
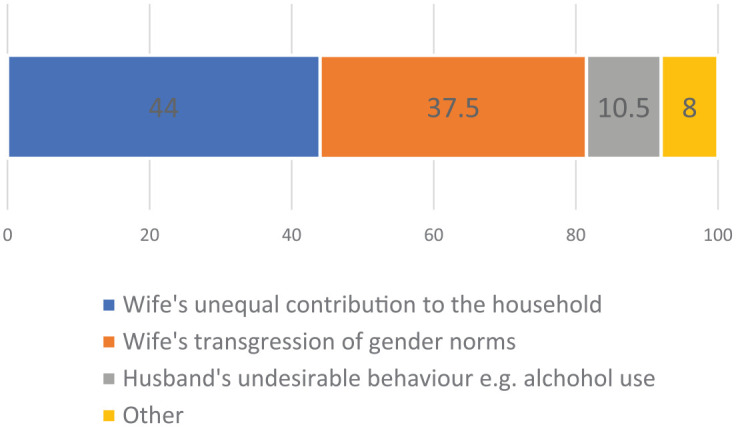
Percentage of directly stated reasons that wife-beating is justifiable (*n* = 47).

The DQ survey (Version 2B) provided information on the socially accepted reasons for husbands to physically assault their wives. Those individuals who indicated that the behavior was acceptable through DQ (*n* = 47) were asked to provide up to three reasons when or circumstances where this form of physical violence is justified. All informants only provided one reason, but responses fell into two main categories relating to (a) inequalities in household resource generation and use (44%), specifically citing women’s relatively lower labor and income contribution and misuse of household resources; and (b) circumstances where women transgress traditional gender norms (37.5%), particularly women’s disobedience (e.g., refusal to run errands for husband) or failure in wifely duties (e.g., preparing dinner on time). Other less frequently cited explanations for wife-beating included the characteristics of the male perpetrator, for example, excessive alcohol use or personality (10.5%). These results are presented in a bar chart in Figure 2.

## Discussion

Using a list randomization experiment (LRE) we find evidence of high but concealed acceptance of physical IPV among subsections of a rural Ethiopian community. Overall, around one third of adults in the community identify wife-beating as justifiable when questioned either directly or indirectly. We find that tolerance of wife-beating is highest among adults with lower levels of education (≤3 years) and among those living in households where economic decision making is controlled by men alone. Furthermore, we identify a discrepancy between direct and indirect question responses, indicating that people who are poorly educated or living in less gender equal households are privately more supportive of wife-beating than they will admit openly to an interviewer (Table 1). Previous studies have suggested that measurement errors and underreporting of IPV may occur in traditional surveys employing direct questioning, due to lack of awareness regarding what constitutes IPV or recall bias (Zegenhagen et al., 2019). Our analyses reveal this underreporting of wife-beating acceptance is due to social desirability bias, the inclination to give socially acceptable answers, in a context where violence between intimate partners has legal and social implications for the perpetrators and victims.

The finding that people who are most likely to justify wife-beating are also more inclined to conceal their views when asked directly is also important for the development of policy and programs working to end IPV. It raises questions about the accuracy of traditional surveys, such as the Demographic and Health Survey, which rely on DQ methods for capturing attitudes to physical IPV among high-risk individuals or communities. It also demonstrates a need for anti-IPV campaigns to encourage a more open dialogue to prevent violence (and its social acceptability) remaining undetected and thus unchanged. This is reflected in words of one traditional leader on the barriers to change “there is not a tradition among the people to openly discuss the conflict between husband and wife. Many people hide the issue, whether it is in practice or beliefs.”

Our results also reveal household poverty is not a good predictor of wife-beating acceptance, which is in line with the inconsistent evidence of a relationship between wealth status and IPV across other parts of SSA (Bamiwuye & Odimegwu, 2014; Semahegn et al., 2019). We find greater support for the idea that improving women’s economic status through paid work or immovable assets can help to prevent IPV (Heise & Kotsadam, 2015), particularly by increasing acceptance of more egalitarian gender norms (Schuler & Nazneen, 2018). The main reason that wife-beating is seen as justified among the Arsi Oromo is women’s lower contribution to the household than men, both in physical farming labor and in income (Figure 2). Encapsulated in the words of one male informant, “She [his wife] doesn’t do what I do for the household.” For our sample, wife-beating is also tolerated under circumstances where women transgress from traditional gender norms (e.g., not following husband’s instructions, refusing to run errands). The significance of underlying gender norms is revealed in one traditional elder’s view on why IPV is tolerated “ . . . it is believed that a man is always above a woman, and woman is always under a man.” For the Arsi Oromo, IPV is conditioned by both gender practices and status concerns. These results highlight the importance of designing interventions that address deep-seated gender norms alongside practical economic needs (Gupta et al., 2013; Svec & Andic, 2018).

We find attitudes in support of wife-beating to reach normative levels (>50% endorse violence toward intimate partners) among men who are less educated and men living in households where they control all economic decision making. Furthermore, we identify that these individuals also attempt to conceal this support when questioned directly, revealing that they are aware that their position on wife-beating is not socially acceptable. Rather than being ignorant of attitude shifts among others in the community (Burszytyn et al., 2018), our results imply that these men may be resistant to or threatened by prevailing attempts to change traditional gender norms. This finding also lends support for the view that increasing inequality and status competition between men may lead some to react against new gender norms, driving the persistence of wife-beating attitudes and behavior across the wider community (Jewkes et al., 2015). That we find pockets of high, but hidden, acceptance of IPV indicates that they should be targeted in future interventions. Reducing wife-beating tolerance among these men may accelerate change in attitude within households (Hayes & Boyd, 2017) and between generations (Semenza et al., 2019).

The relative importance of education versus women’s empowerment in preventing IPV has been widely debated in research and policy (see review in Semahegn et al., 2019). Our analyses identify that education and women’s empowerment act as multipliers in reducing the acceptability of physical IPV. We find that acceptance of wife-beating is entirely absent (0% endorsement) only among those individuals who have both higher levels of education (4+ years of schooling) and live in households where decision making is shared between couples (Table 3). This indicates that educated men, who are also willing to involve women in household decision making, may be less threatened by changing gender norms (Zegenhagen et al., 2019). Furthermore, these results indicate that in addressing low levels of education and unequal gender norms simultaneously, the social acceptability, and thus the occurrence, of IPV could be entirely eradicated. Further studies, employing LRE on larger sample sizes would allow these “low risk” groups to be confidently identified.

Finally, although the results presented here clearly reveal the inaccuracy of traditional direct questioning (DQ) techniques for measuring IPV attitudes and behaviors, the indirect techniques we have developed and used (LRE) also have some limitations. For instance, they are statistically inefficient and require large sample sizes (Gibson et al., 2018), and they fail to consider the possibility of measurement errors in independent variables (Zegenhagen et al., 2019). That said, statistical refinements are underway (e.g., double list design; Moseson et al., 2017), and there is a growing view that these indirect techniques can be further developed and utilized to improve the quality and reliability of IPV data, and to assist with monitoring and evaluation intervention programmes (Peterman et al., 2018).
